# Outcomes of vitrectomy for diabetic tractional retinal detachment in Chicago’s county health system

**DOI:** 10.1371/journal.pone.0220726

**Published:** 2019-08-20

**Authors:** Jared T. Sokol, Sidney A. Schechet, Darin T. Rosen, Kevin Ferenchak, Sherif Dawood, Dimitra Skondra

**Affiliations:** 1 Department of Ophthalmology and Visual Science, University of Chicago, Chicago, Illinois, United States of America; 2 Department of Ophthalmology, Chicago Cook County Hospitals and Health System (CCHHS), Chicago, Illinois, United States of America; Massachusetts Eye & Ear Infirmary, Harvard Medical School, UNITED STATES

## Abstract

**Purpose:**

To examine outcomes of 23-gauge (23G) pars plana vitrectomy (PPV) for complex diabetic tractional retinal detachment (TRD) in Chicago’s Cook County Health and Hospitals System (CCHHS).

**Materials and methods:**

This is a retrospective noncomparative study of diabetic TRD cases that underwent PPV at CCHHS. Primary retinal reattachment rate, visual function, and postoperative complications were analyzed.

**Results:**

Sixty nine consecutive cases were included. Primary reattachment and final attachment were achieved in 68/69 eyes (98.6%). Secondary retinal detachment was noted in 1 eye (1.4%). Vitreous hemorrhage requiring repeat PPV developed in 5 eyes (7.2%) and reoperation due to other complications was required in 4/69 eyes (5.8%). Perfluoropropane (C_3_F_8_) gas tamponade was used in 91.3% of eyes and silicone oil in 8.7% of eyes. Mean LogMAR visual acuity significantly improved from 1.84 ± 0.61 to 0.93 ± 0.66, (*P*<0.0001). Vision was stabilized or improved in 66 eyes (95.7%). Visual acuity of 20/200 or better was achieved in 49/69 eyes (71.0%) and 20/50 or better in 16/69 eyes (23.2%).

**Conclusions:**

Even in patients with severe and advanced diabetic TRD pathology and unique demographics as seen in CCHHS, modern vitrectomy techniques can provide excellent anatomical and visual outcomes.

## Introduction

Tractional retinal detachment (TRD) represents one of the most severe complications and a major cause of vision loss in patients with proliferative diabetic retinopathy (PDR)[1]. Hyperglycemia and molecular events related to diabetes lead to retinal vessel endothelial cell damage, increased vascular permeability, bleeding, retinal vessel occlusion, and subsequently retinal ischemia. An ischemic retina secretes locally active cytokines, such as vascular endothelial growth factor and connective tissue growth factor, which lead to neovascularization and connective tissue formation in the proliferative stage of diabetic retinopathy. This fibrovascular proliferation grows into the vitreoretinal interface and may contract, potentially resulting in TRD[2].

Diabetic TRD is repaired with pars plana vitrectomy (PPV), which allows for simultaneous visualization and manipulation of the retina[3]. In 1981, Michels described posterior membrane segmentation as a surgical technique for the management of TRD by relieving anterior and tangential traction caused by fibrovascular membranes, allowing for reattachment of the retina[4]. The surgical repair of diabetic TRD is among the most challenging surgeries for a vitreoretinal specialist due to the friable nature of the ischemic retina and the presence of extensive fibrovascular membranes[5]. Despite the difficulty of repair, recent advancements in vireoretinal surgery, including smaller gauge instruments, wide angle viewing systems, use of tamponade, self-retaining endoillumination, and the development of medications targeting vascular endothelial growth factor, have greatly improved outcomes in complex TRD cases[6–9].

Cook County Health and Hospitals System (CCHHS) is one of the largest public county hospital systems in the United States and is an important safety net hospital for the city of Chicago. CCHHS serves a primarily underserved population with 43.8% of outpatients using Medicaid as a payor source and 22.4% of outpatients requiring charity care[10]. Furthermore, the patient population of CCHHS is primarily from minority groups, with 52.4% of patients identifying their race as Black and 27.6% of patients identifying their ethnicity as Hispanic or Latino[10].

CCHHS serves patients with severe diabetes and limited access to preventative medical and retinal screening. These patients commonly present with advanced PDR and severe longstanding diabetic TRD with broadly adherent plaques of fibrovascular proliferation. Surgery for diabetic TRD has a high rate of complications postoperatively, especially for eyes with complex pathology. With just a handful of previous studies investigating PPV for diabetic TRD in a county hospital, limited data are available for outcomes of surgical repair in patient cohorts with such demographics and severity of pathology[5,8,11]. The purpose of this study is to examine the visual and anatomical outcomes and complication rates of 23-gauge (23G) PPV for diabetic TRD at CCHHS.

## Material and methods

We conducted a retrospective review of the medical records of all consecutive surgical cases of diabetic TRD performed by a single vitreoretinal surgeon (DS) at CCHHS, between November 2013 and March 2016. Eyes that underwent 23G PPV for primary repair of diabetic TRD and had at least 3 months of follow-up were eligible for inclusion; 8 patients with less than 3 months of postoperative follow-up, or patients with other proliferative vitreoretinal disease, such as proliferative sickle cell retinopathy, were excluded from this study. This study was conducted in accordance with the Declaration of Helsinki. The collection and evaluation of all protected health information was performed in a Health Insurance Portability and Accountability Act (HIPAA)-compliant manner. Ethical approval for this study was obtained from Chicago’s Cook County Health and Hospitals System Institutional Review Board (IRB). Informed consent was not obtained because it was waived by the IRB that approved the study.

23-gauge vitrectomy was performed using the Alcon Constellation vitrectomy machine (Alcon Laboratories, Inc., Fort Worth, TX, USA) using a wide-angle viewing system and scleral depression during the vitreous base dissection. 25 and 27-gauge vitrectomy was not used as it was not available at the hospital where the surgeries were performed. Fibrovascular membrane dissection was performed using a combination of one or more of the following without bimanual technique: the vitrector handpiece, internal limiting membrane forceps, curved scissors, vertical scissors, and/or blunt delaminating spatula. Triamcinolone staining and Perfluoro-n-octane (PFO) liquid was used as needed. Endolaser photocoagulation was applied around retinal breaks and in panretinal photocoagulation (PRP) fashion using a flexible curved illuminated laser. Fluid-air exchange was followed by injection of long-acting tamponade of perfluoropropane gas (C_3_F_8_) (14–16% concentration) or silicone oil. The choice of tamponade was determined by the extent of detachment, the presence and location and size of retinotomy or retinectomy, and the amount of residual traction/fibrovascular membranes. C_3_F_8_ gas tamponade was favored in the vast majority of cases except in eyes requiring large inferior retinectomies and with significant residual traction/fibrovascular membranes. All sclerotomies were sutured. Scleral buckle (SB) was used in cases with significant peripheral vitreoretinal traction or with significant scar tissue that required inferior retinectomy. In patients with a preoperative visually significant cataract, cataract extraction with intraocular lens replacement (CE/IOL) was performed at the time of vitrectomy by a cataract surgeon. To our knowledge, all lens calculations were made using A-Scan ultrasound biometry and measurements of the fellow eye.

During the postoperative period, patients were instructed to maintain face-down positioning for up to 3 weeks at least 80% of the time. Patients were seen weekly for the first 3 weeks after surgery to monitor the postoperative course and reinforce and ensure compliance with instructions. Primary outcome measures included postoperative best-corrected Snellen visual acuity (BCVA), rate of primary reattachment with a single surgery, and rates of postoperative complications including secondary retinal detachment, vitreous hemorrhage needing repeat PPV, anterior hyaloid fibrovascular proliferation (AHFVP), fibrinoid syndrome, neovascular glaucoma, and epiretinal membrane formation (ERM) needing PPV.

BCVA was converted to the logarithm of the minimum angle of resolution (LogMAR) for quantitative comparison. A LogMAR value of 1.98 was used for vision limited to counting fingers (CF) and 2.28 for hand motions (HM)[12]. Vision limited to light perception (LP) or no light perception (NLP) could not be converted to LogMAR, as these do not represent visual acuities, and were thus excluded from this analysis. Preoperative and postoperative LogMAR values were analyzed using a paired, two-tailed *t*-test, and a *P*-value <0.05 was considered statistically significant.

## Results

69 consecutive eyes from 61 patients that underwent 23G PPV for primary repair of diabetic TRD were included in this study. Patient characteristics are described in Table 1. The average age of all patients was 47 years (range, 21–65 years) and average follow-up was 11.4 months (range, 3–28 months). 36 patients were male and 25 were female. 36 patients were Hispanic, 21 were African American, 3 were Caucasian, and 1 patient was Asian.

**Table 1:** Patient characteristics (n = 61).

Patient Characteristics	Number of Patients (%)
Age (years)	
Average	47
Range	21–65
Sex	
Male	36 (59.0)
Female	25 (41.0)
Ethnicity	
Hispanic	36 (59.0)
African American	21 (34.4)
Caucasian	3 (4.
Asian	1 (1.6)
Follow-up Time (months)	
Average	11.4
Range	3–28
HgbA1c (average)	9%

**Fig 1:**
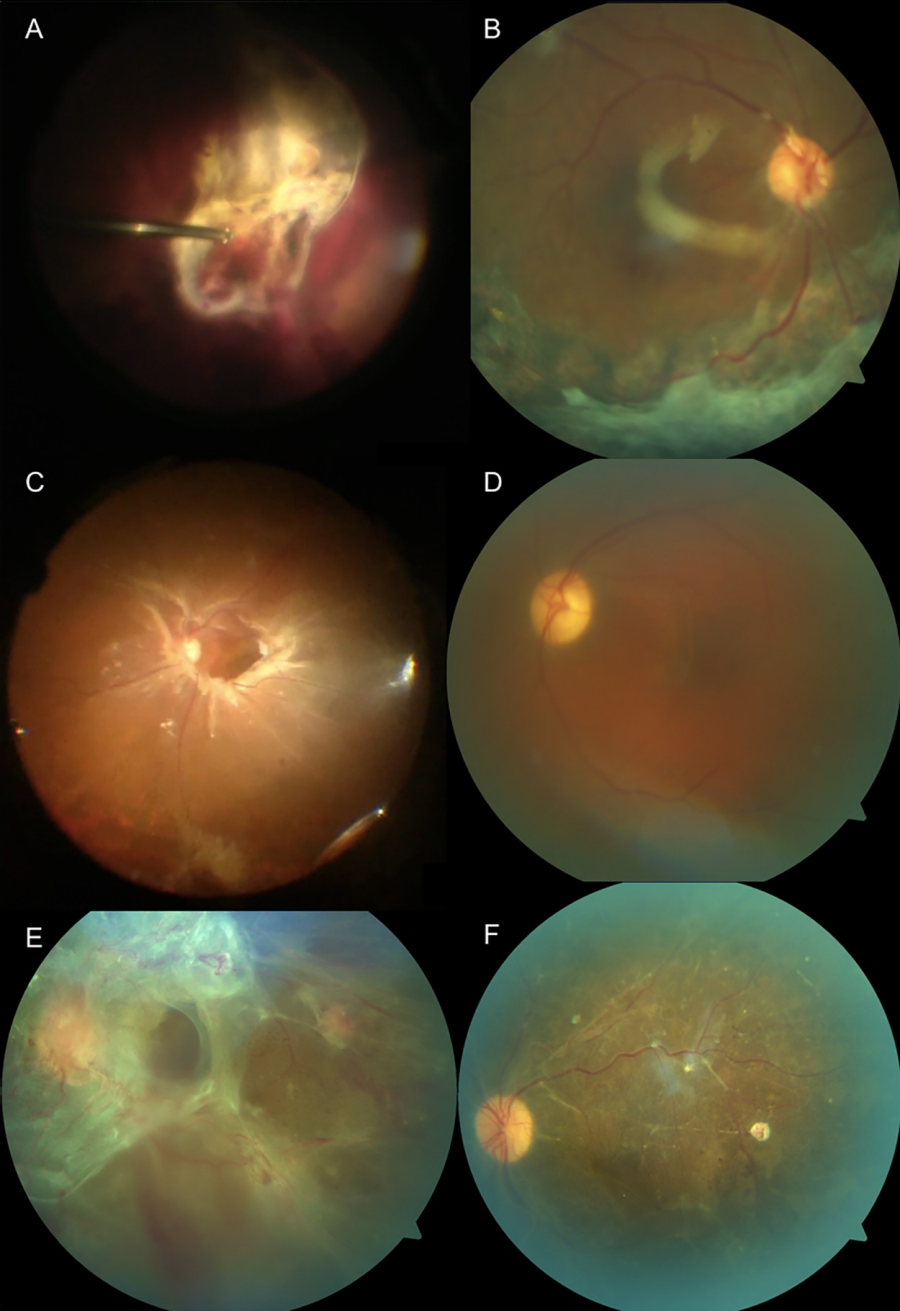
Fundus photographs of advanced diabetic tractional retinal detachments repaired with surgery. (A-B) Intraoperative and postoperative fundus photos in a 50 y/o male with 20-year history of type 2 diabetes mellitus who presented with combined tractional and rhegmatogenous retinal detachments (TRD/RRD) with extensive fibrovascular plaques covering the posterior pole and inferior retina. (C-D) Intraoperative and postoperative fundus photos in a 50 y/o male with 5-year history of diabetes mellitus with combined TRD/RRD and ring of fibrovascular membrane along arcades. (E-F) Preoperative and postoperative fundus photos in a 30 y/o female patient with history of type I diabetes mellitus that presented with chronic macula off TRD with extensive preretinal fibrovascular proliferation. Vision improved from count fingers to 20/80 postoperatively.

69.6% of eyes were characterized by detachments involving the macula, 44.9% by combined tractional and rhegmatogenous retinal detachments (TRD/RRD), and 85.5% by high complexity detachments or severe fibrovascular proliferation (Table 2 along with pre-operative findings, complications, and other anatomic/functional outcomes). TRD/RRD cases included those with preexisting breaks only, a combination of iatrogenic and preexisting breaks, or iatrogenic breaks only. No specific associations were apparent between the presence of retinal breaks and patient age or retinal ischemia. Combined SB was performed in 8/69 eyes (11.6%), combined CE/IOL in 6/69 eyes (8.7%), and combined pars plana lensectomy (PPL) in 3/69 eyes (4.3%), which is summarized in Table 3. Long-acting tamponade was used in all cases, with C_3_F_8_ in 63/69 eyes (91.3%) and silicone oil in 6/69 (8.7%). There were 56 eyes that were phakic at the time of operation, of which 9 underwent combined cataract extraction or lensectomy. Of the 47 eyes that were phakic immediately after vitrectomy, 17 (36.2%) required postoperative CE/IOL in the follow-up period. General anesthesia was used in 96% of cases, monitored anesthesia care (MAC) anesthesia was used in 4% of cases, and mean surgical time was 181 minutes (SD 65.4). Mean post-operative day 1 intraocular pressure (IOP) was 20.0 mmHg (SD 9.3), post-operative week 1 IOP was 15.7 mmHg (SD 4.1), and post-operative month 1 IOP was 15.0 mmHg (SD 4.2). Patients with elevated IOP responed to topical therapy, which was tapered as IOP improved. No patients required surgical intervention for elevated IOP. Fig 1 highlights three cases from the cohort, which effectively represent the typical complexity encountered in the patients analyzed in this study.

**Table 2:** TRD characteristics, anatomical and functional outcomes, and complications (n = 69).

	Number of Eyes (%)
**TRD characteristics**	
TRD only	38 (55.1)
Combined TRD/RRD	31 (44.9)
Macula Involvement	48 (69.6)
High Complexity Detachment or Severe Fibrovascular Proliferation	59 (85.5)
Phakic	56 (81.2)
**Anatomic Outcomes**	
Primary Reattachment with Single Surgery	68 (98.6)
Final Attachment	68 (98.6)
**Visual Outcomes**	
Postoperative BCVA	
> = 20/50	16/69 (23.2)
> = 20/100	38/69 (55.1)
> = 20/200	49/69 (71.0)
> = 5/200	54/69 (78.3)
Better	61/69 (88.4)
Stable	5/69 (7.2)
Worse	3/69 (4.3)
No Light Perception	0 (0)
**Complications**	
Vitreous Hemorrhage	41 (59.4)
Vitreous Hemorrhage Requiring PPV	5 (7.2)
Secondary Retinal Detachment	1 (1.4)
Neovascular Glaucoma	1 (1.4)
Neovascular Glaucoma Requiring Filtering Surgery	0 (0)
Anterior Hyaloid Fibrovascular Proliferation	1 (1.4)
Fibrinoid Syndrome	1 (1.4)
Epiretinal Membrane Requiring PPV	1 (1.4)

TRD, tractional retinal detachment; RRD, rhegmatogenous retinal detachment; BCVA, best-corrected Snellen visual acuity; PPV, pars plana vitrectomy.

**Table 3:** Intraoperative procedures (n = 69).

Surgery Characteristics	Number of Eyes (%)
Combined Scleral Buckle	8 (11.6)
Primary Combined PPL	3 (4.3)
Combined CE/IOL	6 (8.7)
Long-Acting Tamponade	
C_3_F_8_ gas	63 (91.3)
Silicone Oil	6 (8.7)

PPL, pars plana lensectomy; CE/IOL, cataract extraction with intraocular lens implantation.

Retinal reattachment with a single surgery was achieved in 68 of 69 eyes (98.6%) (Table 2). One patient had persistent shallow chronic subretinal fluid in the macula after the primary repair and repeat vitrectomy was performed for draining of persistent submacular fluid. Secondary retinal detachment was observed in 1 eye (1.4%) at 4.5 months postoperatively and was reattached successfully with one additional surgery with combined vitrectomy and lensectomy with scleral buckle and silicone oil tamponade. Final reattachment was achieved in 68 eyes (98.6%). Postoperative vitreous hemorrhage requiring repeat PPV was noted in 5 eyes (7.2%). Although not systematically quanitified, in combined CE/IOL and SB cases inflammation appeared increased. There was one case of neovascular glaucoma that was managed medically and did not require filtering surgery. No eyes developed endophthalmitis, phthisis, corneal decompensation, or secondary angle closure. No patients were noted to have NLP vision and no patients had complications (e.g. IOL positioning) due to prolonged face-down positioning.

Overall, the mean preoperative visual acuity improved from LogMAR 1.84 (SD 0.61, 95% confidence interval (CI) 1.69–1.98) to 0.93 (0.66, 0.77–1.09) after vitrectomy, *P*<0.0001. Significant improvements in postoperative visual acuity were observed in eyes that received C_3_F_8_ but not silicone oil, which is summarized in Table 4. Both eyes with TRD and eyes with TRD/RRD had significant improvement of visual acuity postoperatively (Table 5). Eyes with TRD (55.1%) improved from LogMAR 1.87 (0.58, 1.68–2.05) to 0.78 (0.59, 0.56–0.96), *P*<0.0001, and eyes with combined TRD/RRD (44.9%) improved from LogMAR 1.80 (0.65, 1.57–2.03) to 1.12 (0.70, 0.88–1.37), *P* = 0.00007. Of note, two eyes had preoperative vision that were LP and one eye had postoperative vision that was LP, and therefore could not be converted to LogMAR[12].

**Table 4:** Visual outcomes based on tamponade agent.

Form of Tamponade	Number of Eyes (%)	Mean Preoperative LogMAR (BCVA)	Mean Postoperative LogMAR (BCVA)	*P*-value
C_3_F_8_ Gas	63 (91.3)	1.81 ± 0.62 (~20/1300)	0.87 ± 0.64 (~20/150)	< 0.0001
Silicone Oil	6 (8.7)	2.10 ± 0.45 (~20/2500)	1.52 ± 0.52 (~20/670)	0.11
Total	69 (100)	1.84 ± 0.61 (~20/1400)	0.93 ± 0.66 (~20/170)	< 0.0001

LogMAR, logarithm of the minimum angle of resolution; BCVA, best-corrected Snellen visual acuity

**Table 5:** Visual outcomes based on type of retinal detachment.

Type of Detachment	Number of Eyes (%)	Mean Preoperative LogMAR (BCVA)	Mean Postoperative LogMAR (BCVA)	*P*-value
TRD	38 (55.1)	1.87 ± 0.58 (~20/1500)	0.78 ± 0.59 (~20/120)	< 0.0001
TRD/RRD	31 (44.9)	1.80 ± 0.65 (~20/1250)	1.12 ± 0.70 (~20/270)	0.00007

TRD, traction retinal detachment; RRD, rhegmatogenous retinal detachment; LogMAR, logarithm of the minimum angle of resolution; BCVA, best-corrected Snellen visual acuity.

Improved visual acuity was observed in 61/69 eyes (88.4%). Visual acuity was stabilized in 5/69 eyes (7.2%) and was worse in 3/69 eyes (4.3%). Differences between preoperative and postoperative BCVA are summarized in Tables 2 and 6. Regarding the eyes with stable visual acuity, visual outcomes were limited by macular ischemia/photoreceptor loss due to macula off RD despite retinal reattachment. Of the eyes with worse postoperative acuity, one surgery was complicated by severe hemorrhagic choroidals at the end of surgery as a result of severe bucking due to airway irritation. This patient developed anterior hyaloid proliferation with hypotony and cyclitic membrane 3 months postoperatively and underwent repeat vitrectomy. One patient developed fibrinoid syndrome noted on postoperative day 1, which was managed with systemic and topical steroids without significant response. This patient declined further intervention due to serious comorbidities and eventually developed a white cataract and hypotony. In the third patient, vision was limited by macular ischemia with photoreceptor loss and subsequent dense posterior capsular opacification, ERM, and cystoid macular edema formation under silicone oil for which the patient declined further surgical intervention.

**Table 6:** Visual function comparing preoperative and postoperative visual acuity (n = 69).

BCVA	Number of Eyes (%), Preoperative	Number of Eyes (%), Postoperative
> = 20/50	1/69 (1.4)	16/69 (23.2)
> = 20/200	12/69 (17.4)	49/69 (71.0)
> = 5/200	18/69 (26.1)	54/69 (78.3)

BCVA, best-corrected Snellen visual acuity.

## Discussion

In this study, we report a high rate of anatomic success and low incidence of complications in patients undergoing diabetic TRD repair. The rate of primary reattachment with single surgery and persistent attachment was 98.6% in our series despite the severe pathology seen at CCHHS. Additional vitrectomy was required for postoperative vitreous hemorrhage in 7.2% of cases. Only 5.8% (4/69) of eyes required repeat operation due to other causes including secondary retinal detachment, AHFVP, and ERM formation. Neovascular glaucoma developed in 1 eye (1.4%) but was managed medically without the need for surgery.

Previous studies that have analyzed outcomes of PPV for diabetic TRD have reported a wide range of results. Table 7 provides a detailed review of all relevant literature identified regarding small gauge (23G, 25G, and 27G) vitrectomy outcomes (*n* ≥ 20) in patients with diabetic TRD and TRD/RRD. For increased clarity in presenting previous outcomes data, we decided to group studies before or after 2007, as this appears to be the year when Eckardt’s transconjunctival sutureless 23G vitrectomy technique began to gain popularity over traditional 20G PPV,[13–18]. Before 2007, the overall rate for final retinal reattachment in TRD repair ranged from 53–88%[19–22] and the rate of reoperation was between 16–43% for TRD[19,20,22]. After 2007, the overall rate for final reattachment in TRD repair improved to 67%-100% [5,8,9,23–34] and the rate of reoperation decreased slightly to 6–31% [24,25,28,30,35]. The high reoperation rate both before and after 2007 is likely due to complications that can develop after diabetic vitrectomy. The most frequent complication is postoperative vitreous hemorrhage, which historically had an incidence of up to 75%[36,37], but more recently has been reported in 16–55% of cases [25,26,28,30,35,38]. Despite the large prevalence of this complication, vitreous hemorrhage typically only requires repeat vitrectomy in 5–10% of total cases[5,32,39]. Other common complications include secondary retinal detachment in up to 36% of cases, AHFVP in up to 13% of cases, and fibrinoid syndrome in up to 5% of cases, which represent serious conditions that require repeat vitrectomy and can significantly affect final vision[8],[5,32,40,41],[42]. Neovascular glaucoma occurred in 0–29% of cases before 2007[19–22,43–45] and 0–8% after 2007[5,8,9,18,25–28], with increased incidence in cases with lens removal during vitrectomy[32,46–48].

**Table 7:** Summary of previous literature on small gauge (23G, 25G, and 27G) vitrectomy outcomes (n ≥ 20) in patients with diabetic TRD and TRD/RRD.

Author	Year	# Eyes DTRD^a^	Vitrectomy Gauge and Type	DTRD Pre-op VA (LogMAR unless otherwise noted)	DTRD Post-op VA (LogMAR unless otherwise noted)	Significant DTRD VA improvement (P value)	Tamponade	DTRD Complications	Primary Anatomic Success	Final Anatomic Success
Rahimy et. al[5]	2015	62 (14.5% TRD/RRD)	23G and 20G	2 +/- 0.5	1.4 +/- 0.8	Yes (0.0007)	None/air (37.1%), SO (25.8%), SF6 (19.4%), C3F8 (17.7%)	Secondary RRD (17.7%), NVG (8%)	—	90.3%
Dikopf et. al[32]	2015	70 (70% TRD/RRD)	25G	1.59 +/- 0.88	0.68 +/- 0.77	Yes (<0.001)	SF6 (31.4%), C3F8 (31.4%), BSS (30%), SO (7.1%)	ERM (24%), recurrent vitreous hemorrhage (33%), OHTN (36%), hypotony (3%), NLP (5.7%)	90%	99%
Wang et. al[33]	2016	66 (33.3% TRD/RRD in 4-port group and 26.7% TRD/RRD in 23G group)	4-port bimanual 23G (45%) and 23G (55%)	2.04 +/- 0.76 (4-port), 2.21 +/- 0.67 (23G)	1.16 +/- 0.6 (4-port), 1.31 +/- 0.75 (23G)	Yes (<0.001 in both groups)	C3F8 (58.3% in 4-port and 50% in 23G), SO (41.7% in 4-port and 50% in 23G)	Secondary RD (5.6% 4-port and 10% in 23G), Hypotony (16.7% in 4-port and 3.3% in 23G), NVG (2.8% in 4-port and 6.7% in 23G), reoperation (11.1% in 4-port and 20% in 23G)	94.4% (4-port group), 93.3% (23G group)	100%
Mikhail et. al[34]	2017	109 (29.3% TRD/RRD)	25G	1.17	0.812	Yes (<0.05)	Air (38%), C3F8 (32%), SF6 (23%), SO (7%)	Hypotony (4.6%), OHTN post-op day 1 (10%), NVG (1.8%), recurrent VH requiring vitrectomy (10%)	91%	98%
Storey et. al[8]	2018	403 (21.3% TRD/RRD)	20G, 23G, and 25G	1.73	1.23	Yes (<0.0001)	None (37%), air (7.4%), C3F8 (23%), SF6 (8.4%), SO (24.3%)	Secondary RRD (5.2%), NVG (4.7%), VH post-op day 1 (28%), VH post-op week 1 (33%), VH post-op month 1 (20.9%),	87.60%	92.60%
Shroff et. al[9]	2018	315 (33.9% macular TRD, 45.4% extramacular TRD, 15.2% TRD/RRD, 5.4% FVP with minimal detachment)	23G and 25G	1.67 +/- 0.63	0.78 +/- 0.63	Yes (<0.001)	SF6 and C3F8 (52.7%), SO (25.7%), none (21.6%)	Secondary RD (1.6%), iatrogenic retinal break (52.1%), recurrent VH (18.4%), ERM (7.9%), sclerotomy site break (4.1%), intractable glaucoma (2.5%) endophthalmitis (0.6%)	98.40%	99%
Choovuthayakorn et. al[58]	2019	455 (890–36.6% TRD and 13.5% TRD/RRD of entire cohort)	20G and 23G (46% of TRD and TRD/RRD cases 23G)	44% of eyes improved 2 or more Snellen lines		—	SO (40.4%)	RD (9%)	No DTRD subgroup analysis	

DTRD, diabetic tractional retinal detachment; TRD, tractional retinal detachment; RRD, rhegmatogenous retinal detachment; VA, visual acuity; LogMAR, logarithm of the minimum angle of resolution; SO, silicon oil,; RD, retinal detachment; AHFVP, anterior hyaloidal fibrovascular proliferation; NVG, neovascular glaucoma; VH, vitreous hemmorage; C3F8, perfluoropropane; SF6, sulfer hexafluoride; ERM, epiretinal membrane; OHTN, ocular hypertension; FVP, fibrovascular proliferation.

^a^ all averages and percentages labeled DTRD represent both TRD and TRD/RRD unless otherwise noted.

Studies assessing visual outcomes for TRD repair typically report reaching postoperative visual acuity > = 20/100 in 7%-43% of cases, > = 20/200 in 36.2–62.5% of cases, > = 5/200 in 67–77% of cases, and visual improvement in up to 90% of cases [9,19–23,26,39,43,45,46,49–53]. In our series, 69.6% of eyes were characterized by preoperative macular involvement and 44.9% by combined TRD/RRD, with 55.1% of eyes reaching postoperative acuity > = 20/100, 71.0% of eyes reaching > = 20/200, 78.3% of eyes reaching > = 5/200, and visual improvement in 88.4% of eyes. Despite using tamponade in all cases, only 36.2% of phakic eyes required cataract surgery in the short-term follow-up period in this series. This rate is consistent with other studies describing the need for cataract extraction in 33%-87.1% of vitrectomized eyes for diabetic indications in one to four years postoperatively irrespective of tamponade status[30,54–56]. Visual outcomes in this study seem to be favorable compared to other studies with lower rates of complications, such as secondary retinal detachment and neovascular glaucoma.

It is reasonable to consider that the results of this study could partially be attributed to differences in disease severity or intraoperative technique. However, the patient population in our series represents an underserved community at a large urban county hospital, and is comprised of mostly Hispanic and African-American patients (93%) who frequently present with more advanced PDR[57]. As expected, these demographic and socioeconomic trends are reflected in the high anatomic complexity of these cases. Preoperatively and intraoperatively, many eyes were characterized by combined TRD/RRD (44.9%) and/or macular involvement (69.6%), and over 80% represented severe cases characterized by extensive broad adherent fibrovascular plaques. Thus, the patients in this series likely represent a more complex population than observed in previous studies.

Our overall surgical approach was consistent with many recent studies, but special attention was focused on meticulous removal of fibrovascular membranes and residual hyaloid with triamcinolone staining, which may contribute to both our favorable outcomes and our longer surgical durations [5,7–9]. Full PRP including the anterior retina with scleral depression could contribute to the lower rates of neovascular glaucoma and postoperative vitreous hemorrhage requiring PPV. It is unlikely that the results can be exclusively attributed to minor surgical differences alone since most studies for diabetic TRDs mention thorough removal of membranes and hyaloid and full PRP. Moreover, a distinction in our study was the use of long-acting tamponade in all cases, in particular gas tamponade with C_3_F_8_ 14–16% in more than 90%% of cases, and prolonged face-down positioning. In most diabetic TRD studies in the literature, the tamponade is variable, including cases without any tamponade, air tamponade, variable concentrations of sulfur hexafloride (SF_6_) and C_3_F_8_, or silicon oil and information regarding patient positioning is rarely addressed.

Currently there are no strict guidelines for the use and the type of tamponade (short versus long-acting and gas versus silicon oil) or face-down positioning after vitrectomy for diabetic TRD repair. In this series, we favored the use of long-acting C_3_F_8_ rather than silicone oil, limiting the use of silicone oil mostly to cases involving large inferior retinectomies. In this study, we observed a significant improvement in BCVA in eyes that underwent C_3_F_8_ tamponade but not silicone oil. However, the six eyes that received silicone oil had worse preoperative BCVA and were selected to receive silicone oil tamponade due to their severe pathology needing large inferior retinectomies. This study is not randomized so definitive conclusions for the use of silicone oil versus gas cannot be drawn. Since C_3_F_8_ was used in more than 90% of the cases, our data indicates that excellent outcomes can be achieved by using long-acting C_3_F_8_ in complex TRD cases, even those with severe FVP and combined TRD/RRD.

Strengths of this study include the large population of eyes with complex diabetic TRD operated on by a single surgeon with consistent techniques at a single institution with unique demographics. Limitations of this study include the retrospective design, lack of grading of vitreous hemorrhage, lack of retinal break descriptions, potential for loss to follow-up bias due to the wide range of follow-up times (3–28 months), and the inability to make definite conclusions about tamponade agent and the need for prolonged face down positioning. Although our data suggests that long-acting C_3_F_8_ with prolonged face-down positioning may contribute to improved outcomes, the lack of randomization for the type of tamponade and duration of positioning and the absence of a control group do not allow us to make a definite conclusion.

In conclusion, our findings show that 23G vitrectomy can provide improved outcomes in patients with complex TRD pathology as described in our study representing Chicago’s urban population in a large public hospital. Despite our efforts to prevent late-stage diabetic retinopathy and TRD, many patients are unfortunately faced with these debilitating conditions. Although medical and surgical advancements have greatly improved treatment modalities, further research is warranted to better elucidate which techniques lead to the best outcomes for our patients.

## Supporting information

S1 Data Pre-operative and final visual acuities. All data used to calculate means, standard deviations, confidence intervals, and P values in manuscript.
(XLSX)
